# The long-term effect of being treated in a geriatric ward compared to an orthopaedic ward on six measures of free-living physical behavior 4 and 12 months after a hip fracture - a randomised controlled trial

**DOI:** 10.1186/s12877-015-0153-6

**Published:** 2015-12-04

**Authors:** Kristin Taraldsen, Pernille Thingstad, Olav Sletvold, Ingvild Saltvedt, Stian Lydersen, Malcolm H. Granat, Sebastien Chastin, Jorunn L. Helbostad

**Affiliations:** Department of Neuroscience, Faculty of Medicine, Norwegian University of Science and technology, N-7491 Trondheim, Norway; Department of Geriatrics, St. Olavs Hospital, University Hospital of Trondheim, NO-7006 Trondheim, Norway; Regional Centre for Child and Youth Mental health and Child Welfare, Norwegian University of Science and technology, N-7491 Trondheim, Norway; School of Health Sciences, University of Salford, Manchester, M5 4WT UK; School of Health and Life Science, Institute of Applied Health Research, Glasgow Caledonian University, Glasgow, Scotland UK; Clinic for Clinical Services, St. Olavs Hospital, University Hospital of Trondheim, NO-7006 Trondheim, Norway

**Keywords:** Geriatric assessment, Hip fracture, Physical activity, Accelerometers, Activity monitoring

## Abstract

**Background:**

This study is part of the Trondheim Hip Fracture Trial, where we compared free-living physical behavior in daily life 4 and 12 months following hip surgery for patients managed with comprehensive geriatric care (CGC) in a geriatric ward with those managed with orthopedic care (OC) in an orthopedic ward.

**Methods:**

This is a single centre, prospective, randomized controlled trial. 397 hip fracture patients were randomized to CGC (*n =* 199) or OC (*n* = 198) in the Emergency Department with follow-up assessments performed four and 12 months post-surgery. Outcomes were mean upright time, number and length of upright events recorded continuously for four days at four and 12 months post-surgery by an accelerometer-based activity monitor. Missing data were handled by multiple imputation and group differences assessed by linear regression with adjustments for gender, age and fracture type.

**Results:**

There were no group differences in participants’ pre-fracture characteristics. Estimated group difference in favor of CGC in upright time at 4 months was 34.6 min (17.4 %, CI 9.6 to 59.6, *p* = .007) and at 12 months, 27.7 min (13.9 %, CI 3.5 to 51.8, *p* = .025). Average and maximum length of upright events was longer in the CGC (*p*’s < .042). No group difference was found for number of upright events (*p*’s > .452).

**Conclusion:**

Participants treated with CGC during the hospital stay improved free-living physical behavior more than those treated with OC both 4 and 12 months after surgery, with more time and longer periods spent in upright. Results support findings from the same study for functional outcomes, and demonstrate that CGC impacts daily life as long as one year after surgery.

**Trials registration:**

ClinicalTrials.gov, NCT00667914, April 18, 2008

## Background

Hip fracture patients are generally old and frail, and treatment beyond successful surgery is needed to improve rehabilitation outcomes [1, 2]. Recovery of function to a pre-fracture level, particularly mobility, is the ultimate aim for rehabilitation following a hip-fracture [3]. A decline in function is however common and substantial, where a three times larger decline in function during a year has been reported for those who experienced a hip fracture as compared to those who did not [4].

Effects of interventions have typically been shown for physical performance or self-reported function, giving an indication of what patients are capable of doing. Activity monitoring can provide information of what older people actually do during daily life following a hip fracture. One prospective study using activity monitoring did report very low levels of activity two months following the hip fracture [5]. To our knowledge, there are no studies reporting treatment effects from randomized clinical trials on patients’ free-living physical behavior, defined as what a person actually does during daily life.

Hip-fracture patients are characterized by pre-existing comorbidity and physical impairments and are often regarded as frail [1]. This emphasizes that treatment should be holistic and target not only the fracture itself, but also underlying health problems and disease to avoid an unnecessary decline in physical function [2]. Early mobilization is important for regaining of function and previous activity level, regardless of the level of physical frailty [6, 7]. The medical treatment and the hospital care can also optimize rehabilitation outcomes [2], and enable patients to be mobilized early [8].

This study is part of the Trondheim Hip Fracture Trial conducted to investigate the effect of comprehensive geriatric care (CGC) in a geriatric ward for hip-fracture patients as compared with orthopedic care (OC) in an orthopedic ward in the acute phase during hospital stay primarily on mobility, and secondary on other aspects of function and physical behavior [9]. We found that mobility and activities of daily living 4 and 12 months following surgery was better in the CGC group as compared to OC [10]. CGC was also superior to OC for physical behavior, assessed as upright time and number of upright events early after surgery [8]. However, the long-term effects of hospital interventions on free-living physical behavior are unknown.

Measures of free-living physical behavior can provide information about the success of the intervention in terms of the impact on the patients’ daily life. The present study evaluates the impact of CGC on free-living physical behavior four and 12 months after the hip fracture, for patients randomized in the emergency room at admission to hospital to either CGC or OC. We hypothesized that CGC during the hospital stay results in greater long-term improvements in free-living physical behavior as compared with OC. The primary outcome is daily upright time 4 months after the fracture. Secondary outcomes are number of upright events and mean, maximum, median and variability in length of upright events at 4 months, and all physical behavior variables at 12 months.

## Methods

### Trial design

The study was a single center, prospective, randomized controlled trial. The study protocol [11], the content of the intervention, and results on the primary outcome from the study have been published previously [10]. Activity monitoring data were secondary outcomes and were preplanned for a separate publication. Randomization to either CGC or OC was performed in the Emergency Department using a web-based computer program by block randomization with equal block sizes unknown to the investigators. Participants were included from April 2008 to December 2010. Further details of the study design are described elsewhere [9].

### Participants

Participants were included in the trial if they were admitted to the hospital because of a hip fracture, were at least 70 years of age, previously living in their own homes, able to walk 10 m, and able to give informed consent. Exclusion criteria included pathological fractures, multitrauma injuries, and short life expectancy. Short life expectancy was defined as terminal illness where people were not expected to live longer than three months.

### Interventions

The intervention was performed in the acute hospital setting. Randomized patients were moved from the emergency room to receive pre- and post-operative CGC in a geriatric ward or OC in an orthopedic ward. Both groups received the same perioperative treatment by the same group of surgeons. CGC was performed by an interdisciplinary team of specialized health professionals, developing and executing integrated and individualized treatment plans for the hip fracture patients with the emphasis on comprehensive medical assessment and treatment, special focus on mobilization, and early discharge planning [9, 11]. OC included conventional care with traditional in-hospital treatment procedures. The geriatric ward was somewhat better staffed than the orthopedic ward [9, 11]. The primary health-care services were responsible for follow-up of patients in both groups after the hospital stay, where the Orthopedic surgeons followed-up patients in both groups if needed. The intervention has been described in detail elsewhere [11]. There were no significant group differences in type of surgery [10].

### Demographic data

Pre-fracture function was assessed retrospectively by the Nottingham Extended Activities of Daily Living scale (NEADL) (0–66) [12], the Barthel Index (BI) (0–20) [13], and all participants were asked if they lived alone prior to the fracture. Other background variables included age, gender, fracture type (intracapsular or extracapsular) and surgery method.

Data collected at 1, 4 and 12 months after surgery included NEADL, BI, cognitive function assessed by the Mini Mental Status Examination (MMSE) [14], mobility measured by the Short Physical Performance Battery (SPPB) [15], and registrations of dead/alive at 4 and 12 months. Data from the single item “Walk around outside” from the NEADL was used to describe independence in outdoor mobility the past 14 days at 4 and 12 months, classifying those responding “on your own with difficulty” or “on your own” as independent outdoors walkers.

### Outcomes

All participants attending the 4 and 12 month examination were asked to wear activity monitors for the following four days. The activity monitors were body-worn, single-axis accelerometer-based devices (activPAL PAL Technologies Ltd., Glasgow, United Kingdom) attached by a waterproof tape to the front of participants’ non-affected lower thigh. Only data where there was 24 h of continuous recordings from one or more days was included in the analyses.

In this study, the primary outcome was upright time 4 months after surgery, defined as the mean time per day spent in an upright posture. Secondary outcomes from the same activity recordings included number of upright events 4 months after surgery defined as the mean number of transitions from sitting to standing per day, length of upright events defined as the mean, maximum, median and variation (interquartile range, IQR) in length of all upright events during the recording period, and upright time, number of upright events, and the four measures of length of upright events 12 months after surgery.

The manufacturers’ software gives the number and duration of upright (standing and walking) events. Upright time and number of upright events have previously been validated on a similar population of older persons with impaired mobility showing accurate recordings from an in-lab setting [16].

### Sample size

Sample size calculation was performed for the primary outcome in the Trondheim Hip Fracture Trial, which was lower limb function measured by the Short Physical Performance Battery [15] 4 months after the hip fracture [9]. A total of 380 participants needed to be included at the 4 months examination [10]. In total, 174 CGC and 170 OC completed the 4 month examination, of whom a total of 283 completed the activity monitoring.

### Blinding

It was not possible to blind staff that provided the intervention, study participants, or assessors performing the hospital examinations. The majority of assessors during follow-up were blinded to participants’ group assignments. The first data analyzes were performed blinded for group allocation. The final data analyzes were performed unblinded.

### Data analysis

Activity monitoring data was analyzed using the software version 6.4.1 from PAL Technologies Ltd. The activPAL classifies body postures and provides information about start and end time of sitting/lying, standing and walking events. A single Excel (Office Excel version 11.0, Windows XP Professional, Microsoft, 2003) spreadsheet for each participant was created, using the activPAL software, containing detailed information about event data for all recording days per participants. A custom written MATLAB (MATLAB version 7.1. The MathWorks Inc., Natick, MA, 2005) program derived event information about upright time (time in standing and walking posture), number of upright events (number of changes from sit/lie to standing posture), and length of all upright events from all days with complete 24 h of recordings. Mean upright time (minutes), upright events (numbers), mean event length (minutes), maximum event length (minutes), median event length (minutes), and variation measured as interquartile range (IQR) of event length (minutes) per day were included in the statistical analysis performed in SPSS (SPSS Statistics for Windows, Version 19.0. Chicago: SPSS Inc.). Upright time and upright events for each hour during the day were used to illustrate activity profiles for the two groups.

### Statistical methods

In total 143 participants in the CGC, and 140 in the OC, completed the activity monitoring at 4 months. At 12 months, 135 in the CGC and 117 in the OC completed the activity monitoring, meaning that 29 % and 36 % of the included participants missed activity monitoring data at 4 and 12 months. Participants with complete activity monitoring data had significantly higher pre-fracture scores on the NEADL (mean 44.5 vs 35.8 at 4 months, 45.5 vs 36.0 at 12 months; *p*’s < 0.001) and BI (mean 18.4 vs 17.5 at 4 months, 18.5 vs 17.5 at 12 months; *p*’s < 0.002) compared to participants with incomplete data or without activity monitoring data. Therefore missing data was not randomly distributed.

As a result, missing data were handled by using multiple imputation (MI) [17], using 100 imputed data sets. The imputation model included all variables to be used in the analysis, as well as pre-fracture, 1, 4 and 12 month BI; pre-fracture, 1, 4 and 12 month NEADL; 1, 4 and 12 month MMSE; 5^th^ post-surgery day, 1, 4 and 12 month SPPB, pre-fracture, and dead/alive at 4 and 12 month. Differences in activity outcomes between the two groups at 4 and 12 months were assessed by linear regression analysis, with adjustments for gender, age and fracture type. We also performed a complete case analysis (that is, using the original data without imputed values) as a secondary analysis.

Analyses were carried out in SPSS Inc. (SPSS Statistics for Windows, Version 19.0. Chicago: SPSS Inc.). Ninety-five percent confidence intervals (CI) are reported where relevant. *p* < 0.05 was regarded as statistical significance.

### Ethics

This study complies with the ethical rules for human experimentation as stated in the Declaration of Helsinki. The study was approved by the Regional Committee for Medical Research Ethics (REK 4.2008.335), the Norwegian Social Science Data Service (NSD19109), the Norwegian Directorate of Health (08/5814), and was registered in clinical trials (NCT00667914). Participants signed an informed consent at inclusion. We did not exclude patients with dementia or delirium. In cases where participants were not able to give informed consent, the next of kin was approached. This was approved by the ethical committee.

## Results

**Fig. 1:**
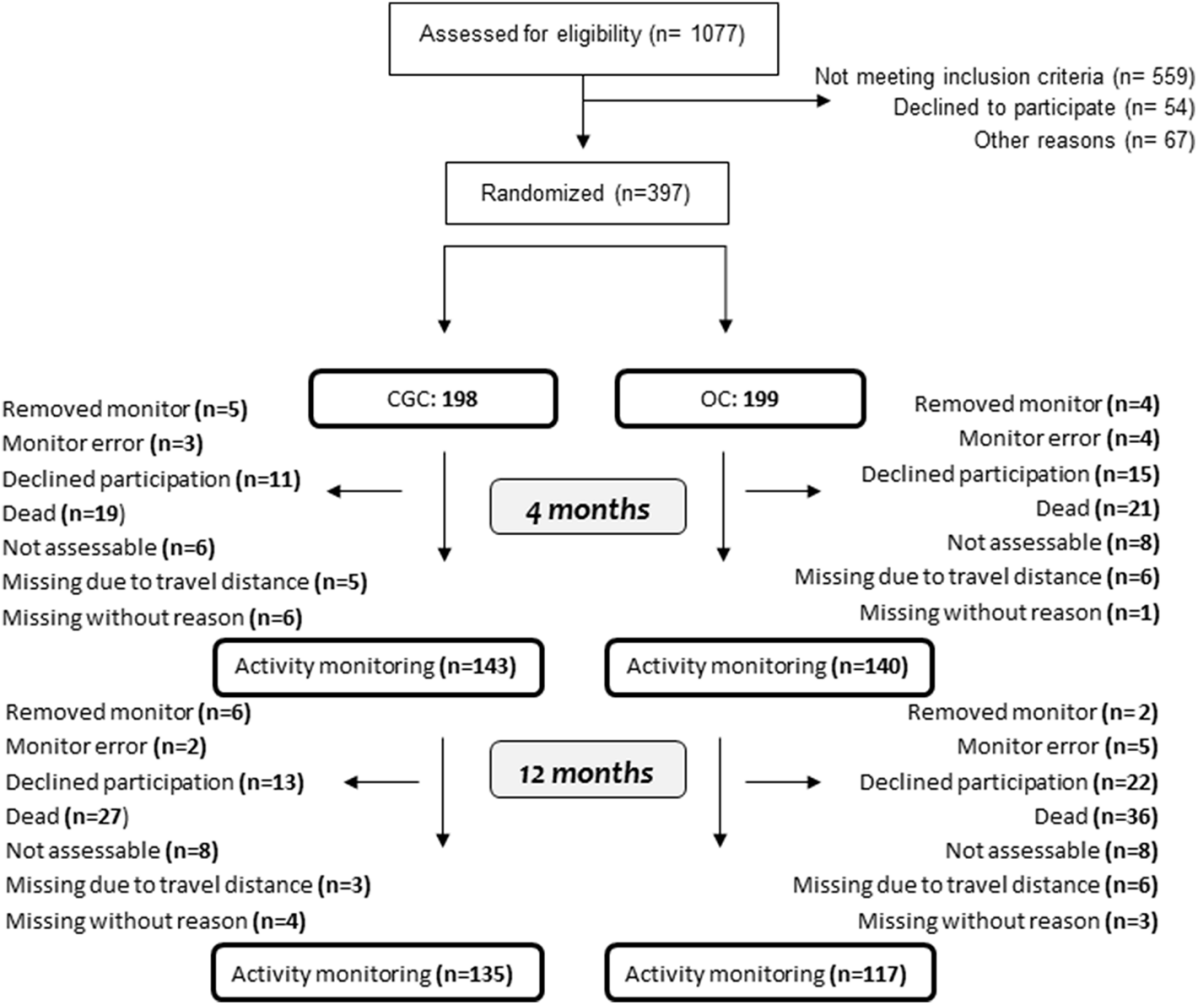
Flow diagram activity monitoring in the Trondheim Hip Fracture Trial. *Note:* The figure shows number of completers at 4 and 12 months. Reasons for drop outs are listed separate for 4 and 12 months, where the total number of drop outs at 12 months lists all drop outs during the 12 months follow-up

397 participants were randomized to receive CGC (*n* = 198) or OC (*n* = 199), of whom a total of 283 and 252 wore activity monitors for a minimum of 24 continuous hours at the 4 and 12 month examination. The flow diagram (Fig. 1) shows the number of participants who completed the activity monitoring and reasons for missing data at 4 and 12 months.

Pre-fracture characteristics were similar in the two groups (see Table 1). Participants’ mean pre-fracture NEADL score was 42 and the BI was 18 and the. Mean age was 83.3 years (SD 6.0, range 70–97), about 74 % were women, and 60 % were living alone. The mean length of hospital stay for the CGC group was 12.6 days (SD 12.6), as compared with 10.4 days for OC (SD 7.0). As previously reported there was no baseline group difference in comorbidity status. The CGC group had on average 5.6 hospital days post discharge and the OC group had 8.4 days. At 4 months, independency in outdoor walking was reported for 49.6 and 63.2 % in the CGC and OC group, respectively. At 12 months, 42.1 and 51.8 % of the CGC and OC group reported that they had been walking independently outdoors. More demographic details and health-care use the first year after hip fracture in the two treatment groups are published in the primary paper and supplementary material from this study [10].

**Table 1:** Pre-fracture status and fracture type

	CGC	OC
	*n* = 198	*N* = 199
NEADL (0–66; mean, SD)	42.5 (17.7)	41.9 (17.5)
BI score (0–20; mean, SD)	18.3 (2.3)	18.1 (2.8)
Age (mean, SD)	83.4 (5.4)	83.2 (6.4)
Females (*n*, %)	145 (73.2)	148 (74.4)
Living alone (*n*, %)	115 (58.1)	124 (62.3)
Intra capsular fracture (*n*, %)	119 (60.1)	127 (63.8)
(hemi arthroplasty, *n*, %)	76 (63.9)	89 (69.3)*
Extra capsular fractures (*n*, %)	79 (39.9)	72 (36.1)*
(bone plates and screws, *n*, %)	67 (84.8)**	63 (87.5)**
Other (*n*, %)	15 (19.0)**	9 (12.5)**

*Including one participant with an extracapsular fracture operated with a hemi arthroplasty

**Including participants treated with combination of surgery or no surgery at all

Notes: BI = Barthel Index; NEADL = Nottingham Extended Activities of Daily Living Scale

### Free-living physical behavior

**Fig. 2:**
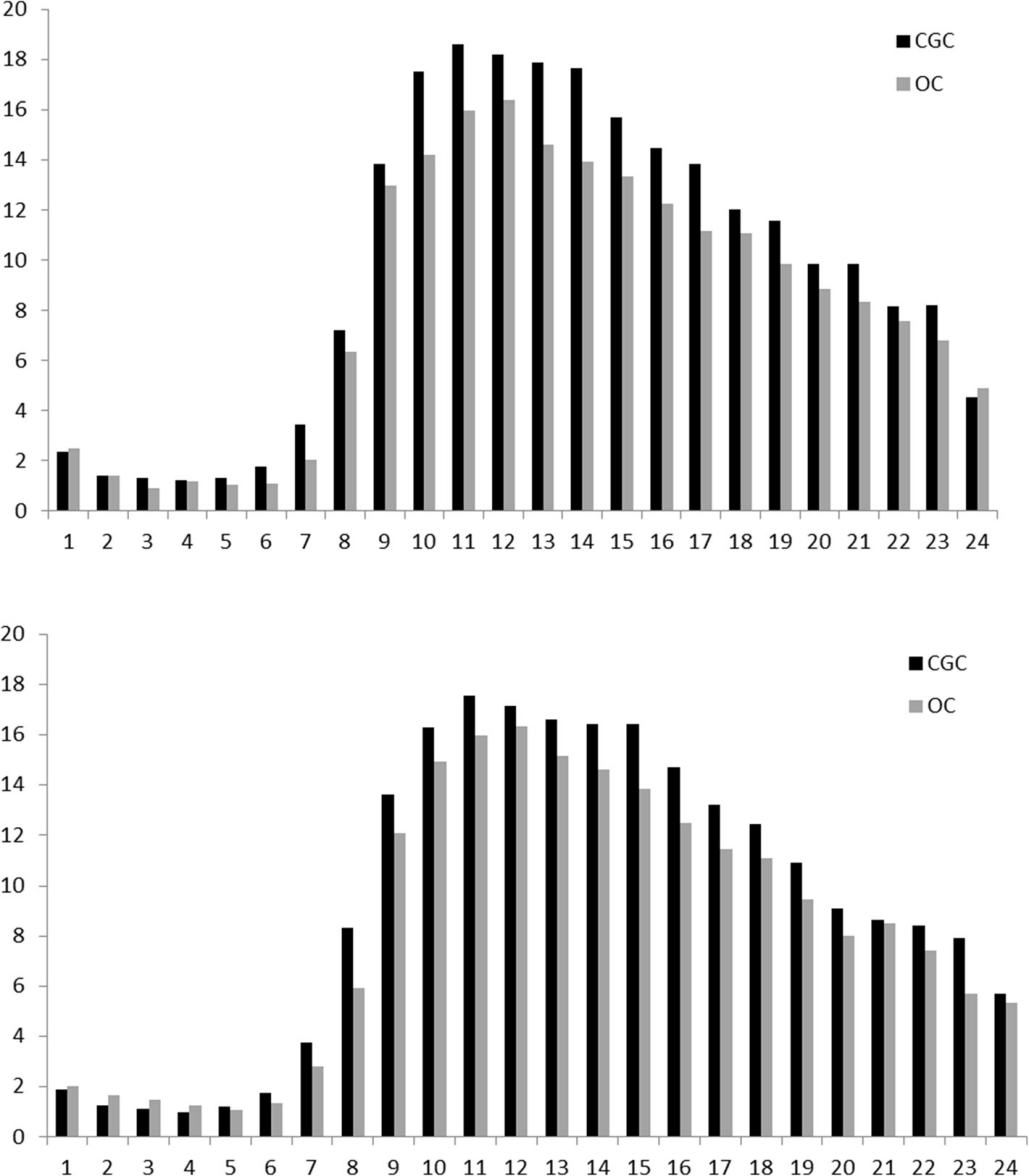
Upright time over 24 h 4 months (upper) and 12 months (lower) postsurgery, showing the mean time in upright for each hour of the day for comprehensive geriatric care (CGC) and orthopedic care (OC)

Using multiple imputation analysis, the level of physical behavior measured as mean daily upright time was better in the CGC group at 4 months, with 231.9 min in the CGC and 198.3 min in the OC, with an estimated group difference of 34.6 min (CI 9.6 to 59.6, *p* = 0.007) representing 17.4 % of mean upright time performed in the OC group. At 12 months the CGC group spent in mean 230.9 min and OC spent 199.5 min upright, with estimated group difference of 27.7 min (CI 3.5 to 51.8, *p* = 0.025) representing 13.9 % of mean upright time in the OC group. The CGC group had longer average duration (*p*’s < 0.033) and longer maximum length (*p*’s < 0.042) of upright events as compared to the OC group. Results are presented in Table 2. There were no statistically significant group differences for number of upright events at either 4 or 12 months, and the median length and variation in length of upright events was also not different between groups. The diurnal variation in upright time is shown in Fig. 2, demonstrating similar patterns for the two groups with a peak around 11–12 a.m.

**Table 2:** Measures of Physical Behavior 4 and 12 months after Hip Fracture

	Descriptive statistics Based on complete cases			Estimated group difference Based on multiple imputation
	Total sample	CGC	OC	Difference
	Mean (SD)	Mean (SD)	Mean (SD)	CGC-OC; CI (*p*-value)
4 months				
*n*=	283	143	140	397
Upright	215.3 (136.9)	231.9 (138.1)	198.3 (134.1)	34.6; 9.6 to 59.6 (.007)*
No. of Events	44.5 (21.3)	44.9 (19.1)	44.0 (23.5)	1.8; 2.8 to 6.3 (.452)
Event length:				
Mean	4.7 (2.7)	5.1 (2.6)	4.4 (2.9)	0.6; 0.1 to 1.2 (.019)*
Max	44.4 (35.5)	48.0 (34.1)	40.7 (36.6)	7.2; 0.3 to 14.1 (.042)*
Median	2.5 (1.3)	2.6 (1.2)	2.3 (1.3)	0.2; 0.1 to 0.5 (.137)
IQR	4.7 (3.3)	5.0 (2.9)	4.5 (3.6)	0.5; 0.1 to 1.2 (.100)
12 months				
*n*=	252	135	117	397
Upright	216.3 (134.2)	228.4 (138.3)	202.3 (128.4)	27.7; 3.5 to 51.8 (.025)*
Events	43.8 (19.9)	43.9 (18.7)	43.8 (21.3)	0.2; 4.6 to 5.1 (.930)
Event length:				
Mean	4.8 (2.8)	5.1 (2.6)	4.5 (2.9)	0.6; 0.1 to 1.1 (.033)*
Max	48.2 (35.1)	51.2 (36.0)	44.7 (33.9)	7.2; 0.5 to 13.9 (.046)*
Median	2.4 (1.3)	2.5 (1.1)	2.4 (1.4)	0.1; 0.2 to 0.4 (.420)
IQR	4.9 (3.4)	5.1 (3.1)	4.6 (3.8)	0.5; 0.2 to 1.1 (.159)

*Statistical significant group differences (*p* < 0.05)

*Notes:* CGC-OC = unstandardized estimated B; Upright = time in upright position (min); Events = number of upright event; Event length = Mean, Maximum, Median and variation (IQR) in length of upright events

Complete case analysis for upright time gave slightly different results at both 4 and 12 months (including 283 and 252 as compared to 397 and 397 in the MI analyses), with a group difference in mean upright time of 31.6 min (CI 1.0 to 62.2, *p* = 0.043) and 28.7 min (CI 3.6 to 61.0, *p* = 0.082) respectively. The complete case analysis also gave slightly different results for mean (group difference of 0.6, CI 0.004 to 1.2, *p* = 0.048, and group difference of 0.7, CI 1.0 to 62.2, *p* = 0.050) and maximum (group difference of 7.0, CI 1.1 to 15.1, *p* = 0.091, and group difference of 7.2, CI 1.5 to 15.8, *p* = 0.104) length of upright events. Number of upright events, median and variation in length of upright events did not differ notably between the complete case and MI analyses, neither for the 4 nor the 12 months results.

### Adverse event management

The trial was carried out as planned according to the protocol [11]. Treatment was offered in parallel in the two treatment arms, where surgery and follow-up of new incidences including adverse events after discharge were offered for both groups by the orthopaedics and the primary health care system as standard routine.

## Discussion

This study assessed effects of acute hospital treatment of hip fracture patients on objectively monitored free-living physical behavior 4 and 12 months after surgery. The potential long-term effects of CGC treatment during the hospital stay was compared with traditional OC. We found that patients treated with CGC were upright for longer time periods at both 4 and 12 months after hip-fracture surgery, with the estimated group difference of 35 min per day at 4 months and 28 min at 12 months, representing 17 and 14 %, of mean upright time in the OC group, respectively.

This is, to our knowledge, the first study to monitor free-living physical behavior the first year following a hip fracture and to evaluate effects of hospital treatment on an objectively measured outcome directly related to participants’ daily life. Results presented in this paper confirm the positive effect of hospital intervention on functional outcomes presented in the main paper from the study [10].

We have previously shown that CGC patients, as compared to OC patients, did spend more time in upright on the 4^th^ day post-surgery, indicating a different treatment approach in the geriatric ward compared to the orthopedic [8]. The CGC delivered by an interdisciplinary team of specialized health professionals had particular emphasis on comprehensive medical assessment and treatment, mobilization and early discharge planning [11]. Together this approach was important for the hip-fracture patients’ long-term recovery after the surgery. As described by Clegg and colleagues (2013) [18], some older persons are more vulnerable, and this could perhaps explain why a stressor like a hip fracture may cause more serious changes in function and activity levels if the treatment approach is not optimal in the early phase after the fracture. Our study has demonstrated that treatment with CGC during the hospital stay is beneficial for older persons after hip fracture, in line with recommendations for treatment for other acutely sick, hospitalized geriatric patients [19].

The group difference in upright time was not shown for number of upright events, indicating that the length of upright events increased and not the number of these events in the CGC relative to the OC group. The diurnal variation of upright time was similar for both groups. A similar diurnal variation for upright time was also shown for the community-dwelling older persons in the study from Grant and co-workers (2010) [20], with a peak around noon followed by a decline the following hours. During the hospital stay, on day four post-surgery, both upright time and number of upright was greater in CGC as compared to OC [8], indicating that when patients’ activity levels are really low, differences are seen for both these measures as well as the diurnal variation. The group difference shown at 4 and 12 months, that more upright time was not followed by an increase in upright events, indicating that the length of upright events increased with no change in the pattern itself. In this study we included four measures of length of upright events, and found that it was the average and maximum length of the upright events that was larger for CGC, indicating that CGC participants recovered more freedom of movement and elasticity in their behavior as compared to the OC participants.

Rehabilitation following hip fracture should aim to regain sufficient function so that people can live as they did prior to the fracture [2]. Better physical function for the CGC group was shown by Prestmo et al. (2015) [10] and in the present study we found longer upright time. However, we cannot conclude with a causal explanation for the relation between improved physical function and free-living physical behavior in the CGC group. From other research we do know that a carry-over effect of improved function into physical behavior could be a challenge. One study on patients following a total hip or knee arthroplasty, showed that participants did not adopt a more active behavior although the other variables of functioning improved [21]. In our study, the CGC had more upright time early after surgery [8] and better free-living physical behavior at 4 and 12 months, confirming the importance of optimal hospital treatment including mobilization to be able to be physically active long term after a hip fracture.

One of the strengths of the present study is that we have objective information about free-living physical behavior in this sample of frail people on 71.3 % of participants at 4 months and 63.5 % at 12 months. The unsuccessful blinding of assessors is a limitation for the performance-based tests in this study, whereas the activity monitoring is believed to be relatively unaffected. Free-living physical behavior represents what persons actually do during their daily life, providing effects highly relevant for the participants. Most missing data are related to the health status of the participants, like deaths, declined participation, not assessable and removed monitors. There were however somewhat more missing data in the OC arm at the 12 months assessment, mainly due to more deaths in this group (36 versus 27 in the CGC arm) and more people who declined to participate (22 versus 13 in the CGC arm), which may also be related to the better effect of the intervention in the CGC arm. The high number of missing data is the most important limitation with the study, where those not completing the activity monitoring had lower function. The statistical method used in this study strengthens however the results. Data were not missing completely at random, and the complete case analyses are expected to give biased results, while the MI analyses are expected to give less bias.

## Conclusion

Patients treated with comprehensive geriatric care in a geriatric ward during hospital stay spent more time upright both 4 and 12 months after surgery, confirming a beneficial long term impact of acute CGC on participants’ free-living physical behavior. This study confirms that hip-fracture patients can benefit from CGC in the acute phase, with impacts on patients’ daily life up to one year after surgery, as compared to traditional OC in an orthopedic ward. The group difference was shown only for length rather than number of upright events. Although the diurnal patterns of upright time were similar, the CGC spent longer periods being upright with longer average and maximum length of the upright periods as compared to OC.

## Availability of data

The activity monitoring data files for the single participants are not shared due to participant confidentiality.

## Abbreviations

BI: the Barthel Index
CGC: comprehensive geriatric care
IQR: interquartile range
MMSE: the Mini Mental Status Examination
NEADL: the Nottingham Extended Activities of Daily Living scale
OC: orthopedic care
SPPB: the Short Physical Performance Battery
